# Supplementary material for: Pharmaceutical Residues in Senior Residences Wastewaters: High Loads, Emerging Risks
Source: Molecules. 2021 Aug 20;26(16):5047. doi: 10.3390/molecules26165047 (PMC8399164; doi:10.3390/molecules26165047)
Supplement: Supplementary file 1 [file molecules-26-05047-s001.zip › molecules-1306616-SI.pdf]

molecules

MDPI

SUPPLEMENTARY INFORMATION

Article

Pharmaceutical Residues in Senior Residences Wastewaters: High Loads, Emerging Risks

Silvia Lacorte [1,*], Cristian Gómez-Canela [2] and Carole Calas-Blanchard [3,4]

[1] Department of Environmental Chemistry, IDAEA-CSIC, Jordi Girona 18-26, 08034 Barcelona, Catalonia, Spain
[2] Department of Analytical Chemistry and Applied (Chromatography section), School of Engineering, Institut Químic de Sarrià-Universitat Ramon Llull, Via Augusta 390, 08017 Barcelona, Spain; cristian.gomez@iqs.url.edu
[3] Biocapteurs-Analyses-Environnement, Université de Perpignan Via Domitia, 52 Av Paul Alduy, CEDEX, 66860 Perpignan, France; carole.blanchard@univ-perp.fr
[4] Laboratoire de Biodiversité et Biotechnologies Microbiennes, USR 3579 Sorbonne Universités (UPMC) Paris 6 et CNRS Observatoire Océanologique, 66650 Banyuls-sur-Mer, France
* Correspondence: silvia.lacorte@idaea.csic.es or slbqam@cid.csic.es

Citation: Lacorte, S.; Gómez-Canela, C.; Calas-Blanchard, C. Pharmaceutical Residues in Senior Residences Wastewaters: High Loads, Emerging Risks. *Molecules* **2021**, *26*, 5047. https://doi.org/10.3390/molecules26165047

Academic Editor: James Barker

Received: 4 July 2021
Accepted: 12 August 2021
Published: 20 August 2021

Publisher's Note: MDPI stays neutral with regard to jurisdictional claims in published maps and institutional affiliations.

Copyright: © 2021 by the authors. Licensee MDPI, Basel, Switzerland. This article is an open access article distributed under the terms and conditions of the Creative Commons Attribution (CC BY) license (http://creativecommons.org/licenses/by/4.0/).

Table S1. Method Detection Limits (MDL, μg/L) of the SPE and LC-MS/MS methodology used.

Compounds	MDL (μg/L)
Dichlorobenzyl alcohol	0.012
Amoxicillin	0.036
Amylmetacresol	0.258
Acetylsalicylic acid	0.092
Atorvastatin	0.004
Bicalutamide	0.003
Budesonide	0.041
Caffeine	0.004
Carbamazepine	0.003
Chlormethiazole	0.007
Chlorpheniramine	0.018
Clarithromycin	0.010
Cloperastine	0.007
Cyclophosphamide	0.003
Dextromethorphan	0.001
Diclofenac	0.010
Donepezil	0.007
Dutasteride	0.001
Escitalopram	0.009
Estrone	0.026
Ezetimibe	0.001
Fluticasone	0.002
Furosemide	0.008
Gabapentin	0.007
Ibuprofen	0.118
Ifosfamide	0.007
L-Ascorbic Acid	0.132
Levetiracetam	0.072
Levofloxacin	0.010
Lidocaine	0.004
Macrogol	0.123
Megestrol	0.001
Mycophenolic ac.	0.002
Paracetamol	0.026
Prednisone	0.026
Pregabalin	0.179
Quetiapine	0.011
Rosuvastatin	0.001
Sulfamethoxazole	0.019

Tiotropium	0.003
Trazodone	0.004
Valsartan	0.006
Vildagliptin	0.009

Table S2. Concentration (µg/L) of pharmaceuticals in SP 1. For clarity, shaded areas indicate values below MDL.

	SP 1														
	15/05/2017			16/05/2017			17/05/2017			18/05/2017			19/05/2017		
**Compounds**	**9h**	**13h**	**16h**	**9h**	**13h**	**16h**	**9h**	**13h**	**16h**	**9h**	**13h**	**16h**	**9h**	**13h**	**16h**
Dichlorobenzyl alcohol				1.71	46.7	0.79	1.63	0.62	32.2				1.02	1.82	2.40
Amoxicillin	0.14			0.08	0.04		0.32	272	0.12					27.5	
Amylmetacresol				10.4	6.48	2.54	4.77	0.70	4.85	6.82		0.88	9.19	9.61	31.0
Acetylsalicylic acid	88.0	52.5	62.3	2891	194		179	288	365	298	24.7	13.6	86.5	581	687
Atorvastatin	0.01	0.01	0.09	0.01	0.02	0.02	0.03	0.03	0.81	0.02	0.02	0.06	0.09	0.36	0.01
Bicalutamide									0.22						0.44
Budesonide	0.44							0.58	0.22					0.14	0.11
Caffeine	23.4	22.6	3.38	29.8	14.2	30.2	8.27	28.5	43.6	5.83	1.85	3.83	9.88	21.5	99.6
Carbamazepine	1.39	3.71	0.07	5.38	6.11	0.75	3.34	0.20	8.50	4.84	0.65	1.76	1.16	78.7	22.0
Chlormethiazole	77.0	7.18	0.15	11.7	84.6	44.0	5.21	0.09	149	24.9	0.18	0.73	0.33	5.33	10.2
Chlorpheniramine		0.05		0.32	0.06		0.02	0.23	0.14	0.47	0.03	0.02		0.02	0.04
Clarithromycin						0.07		0.03				0.01			
Cloperastine															
Cyclophosphamide	5.10	0.50	0.05	0.63	0.14	0.09	3.75		0.22	4.19	0.18	0.03	7.51	26.3	0.55
Dextromethorphan	0.01		0.04	0.09				0.01	0.01						
Diclofenac	1.07	0.54	0.06	1.29	0.20	2.19	4.67	0.06	3.03	7.30	0.11	0.03	1.75	17.1	2.74
Donepezil	0.01				0.02			0.03							
Dutasteride															
Escitalopram	0.06	1.23	0.04	0.12	0.08		0.10		0.05		0.08	0.06		0.07	0.30
Estrone							1.08		5.34						
Ezetimibe	1.13				0.04		0.08							0.08	
Fluticasone			0.03												
Furosemide	0.74	1.25	0.03	1.68	1.06	0.01	0.80	0.12	2.50	3.19	0.42	0.84	0.36	2.24	10.6
Gabapentin		0.26		0.25	1.03	0.77	0.04		0.49	0.19	0.10	0.13	0.37	2.09	0.11
Ibuprofen		1.93		7.18	15.8	5.64	67.0	87.5	83.8	0.20		1.04	2.04	54.9	20.6
Ifosfamide															
L-Ascorbic Acid		0.43	0.19	0.27	0.18	0.33	0.21	0.26		3.54	0.33	0.55	0.23	0.50	

Levetiracetam				4.48	44.4	6.38			13.00	4.93	0.25	0.22	50.9		
Levofloxacin	7.19	1.83	0.67	63.6	2.21	0.08	7.57	6.16	11.6	56.2	6.42	1.44	0.54	29.8	44.7
Lidocaine	0.22									0.01			0.03		
Macrogol	47.6	49.1	42.0	366	8.12	146	47.2		29.4	25.5	5.39	12.8	74.4	30.7	37.9
Megestrol	0.03	0.22	0.06	0.24	0.55	0.03	0.02	0.03	0.17	0.03	0.16	0.18	0.30	1.61	0.64
Mycophenolic ac.	0.13	0.13	0.07	7.55	1.14	0.09	0.26	0.24	288	1.98	4.70	1.26	0.08	2.51	41.0
Paracetamol	125	236	11.2	775	299	165	218	54.6	461	259	29.4	21.3	82.4	394	307
Prednisone	0.46			3.04	4.52	0.54	4.94	0.95			0.74			5.38	16.1
Pregabalin	0.25	1.34		6.86	0.60	0.30	1.02	0.44					0.21	0.42	
Quetiapine	5.52	3.08	0.99	13.3	29.5		13.0	2.24	90.9	3.85	19.0	5.78	12.0	44.9	50.4
Rosuvastatin								0.01	0.01			0.01		0.01	
Sulfamethoxazole	0.53		1.15			0.04	0.17		11.6	4.59	0.07	8.08	2.87	116	71.9
Tiotropium															
Trazodone	10.1	16.0	2.43	7.31	6.88		3.55	3.28	10.5		0.20	0.67	1.92	16.3	3.73
Valsartan		0.01	0.07	0.03	0.31	0.01	0.02	0.02	5.83	0.03	0.04	0.02	0.01	3.80	0.22
Vildagliptin															

Table S3. Concentration (µg/L) of pharmaceuticals in SP 2. Shaded areas indicate values below MDL.

	SP 2								
	16/05/2017	17/05/2017	18/05/2017		19/05/2017		22/05/2017		23/05/2017
**Compounds**	**11h**	**15h**	**14 h**	**19h**	**9h**	**17h**	**14h**	**17h**	**14h**
Dichlorobenzyl alcohol			0.40				1.36	1.74	0.62
Amoxicillin	0.15			0.11		0.04	0.47		
Amylmetacresol	20.7	3.94	1.32		9.43	6.78	0.98		3.04
Acetylsalicylic acid	240	248	164	471	185	19.5	193	1126	125
Atorvastatin	0.10		0.13	0.07		0.02	0.01	0.04	0.11
Bicalutamide									
Budesonide									0.42
Caffeine	81.4	18.2	3.03	4.44	13.7	12.4	30.8	30.1	79.7
Carbamazepine	0.18	0.06			0.02	0.04	0.01	0.06	
Chlormethiazole	0.25	0.07			0.01	0.01		0.04	
Chlorpheniramine	0.68	0.19	0.01	0.02	0.26	0.16			
Clarithromycin	0.09								
Cloperastine	0.01	0.01							
Cyclophosphamide	16.6	1.88	1.05	0.44	2.66	2.93	0.56	1.69	0.87
Dextromethorphan									
Diclofenac	1.22	0.02	0.13	0.07	0.69	0.59	0.05	0.34	1.37
Donepezil	0.03	0.02	0.01		0.01				
Dutasteride									
Escitalopram	0.05	4.26	0.12	0.15	0.11	0.02	0.15	1.61	
Estrone					1.02				

Ezetimibe	0.52								
Fluticasone						0.01			
Furosemide	3.44	0.14	0.91	0.26	0.06	0.37	0.05	0.53	0.06
Gabapentin	0.06	0.02	0.04	0.34	0.02	0.09	0.15	0.08	0.01
Ibuprofen	16.6	10.8	1.02	60.6	29.2	8.59		11.93	1.86
Ifosfamide									
L-Ascorbic Acid	0.48	0.20	0.26	0.28	0.21	0.22	0.23		0.20
Levetiracetam	30.8	3.02							
Levofloxacin	1.35	0.37	0.03	0.06	0.53	0.36	0.11	0.27	0.13
Lidocaine				0.01					
Macrogol	108	520	16.6	31.7	197	62.5	598	164	479
Megestrol	0.13	0.06			0.01	0.04			0.02
Mycophenolic ac.	5.39	3.11	0.40	0.52	0.58	0.08	102	145	25.5
Paracetamol	1227	184	33.4	70.8	83.1	89.2	111	108	165
Prednisone					2.38	3.27		5.43	
Pregabalin									
Quetiapine	14.0	23.7	0.68	0.57	1.87	0.90	2.47	12.8	
Rosuvastatin									
Sulfamethoxazole									
Tiotropium	0.02								
Trazodone	31.9	16.1	1.65	1.06	4.04	5.09	2.90	4.71	0.38
Valsartan	0.20	0.01	4.50	1.98	1.22	5.63	1.45	3.30	0.44
Vildagliptin	0.08	0.17					0.12	0.07	0.02

Table S4. Concentration (μg/L) of pharmaceuticals in FR 1. Shaded areas indicate values below MDL.

	FR 1														
	03/07/2017			04/07/2017			05/07/2017			06/07/2017			07/07/2017		
**Compounds**	**9h**	**13h**	**16h**	**9h**	**13h**	**16h**	**9h**	**13h**	**16h**	**9h**	**13h**	**16h**	**9h**	**13h**	**16h**
Dichlorobenzyl alcohol	0.11			0.15			0.18				0.09	0.11	0.11	0.19	0.14
Amoxicillin	2.46			2.18	0.35				0.89	457			0.51		0.38
Amylmetacresol	16.8	1.40	0.89	0.86		2.39	26.1	15.0	16.4	19.4	9.60		2.81	0.66	4.26
Acetylsalicylic acid	0.46	3.10	2.81	1.69	1.62	1.71	0.68	0.60	0.59	0.32		0.47	1.37	0.23	11.0
Atorvastatin	0.58	0.17	0.21	0.04	0.01	0.01	0.04	0.02	0.01	0.01	0.01	0.03	0.01		0.03
Bicalutamide															
Budesonide		0.05	0.11	0.13		0.13		0.12			0.13				0.07
Caffeine	103	37.5	38.9	39.5	20.2	28.6	19.2	22.0	35.4	54.2	32.2	26.0	37.1	23.0	31.3
Carbamazepine	0.07	0.02	0.02	0.03	0.01	0.02	0.03	0.02	0.02	0.02	0.01	0.02	0.02	0.01	0.02

Chlormethiazole															
Chlorpheniramine															
Clarithromycin															
Cloperastine															
Cyclophosphamide	1.22	0.20	0.03	0.20	0.17	0.01	0.21	0.02	0.39	0.01	1.88	1.62	5.26	3.51	0.26
Dextromethorphan															
Diclofenac	0.03	0.02	0.01	0.02	0.07		0.02	0.37		0.07	17.5	0.22	0.02		0.15
Donepezil	0.04	0.01				0.01		0.02		0.02					
Dutasteride															
Escitalopram	0.79	7.39	0.63	0.22	0.12	8.85	0.16	0.11	0.06	0.34	0.02	0.14	0.76		0.61
Estrone		0.09	0.10		0.08	0.04	0.06	0.11	0.39	0.06	0.09	0.08			0.04
Ezetimibe															
Fluticasone															
Furosemide	39.8	7.26	13.1	6.72	0.49	15.5	109	5.68	2.32	0.66	0.24	1.57	8.23	0.06	12.4
Gabapentin															
Ibuprofen	bdl	1.73	52.2	2.56	0.74	9.54	3.38	1.44	4.89	0.91	0.51	1.56	0.11	36.4	2.88
Ifosfamide	6.48	3.42	1.48	5.10	4.39	3.72	10.4	4.98	6.91	11.3	4.85	4.21	4.05	1.56	7.25
L-Ascorbic Acid	17.8	1.35	13.2	14.2	5.20	11.8	9.89	7.00	11.9	2.40	9.89	13.0	5.03	5.34	8.02
Levetiracetam	415	20.8	68.5	22.7	992	2094	33.1	16.7	11.6	23.3	7.06	263	11.7	5.42	48.0
Levofloxacin															
Lidocaine	0.05									0.01					
Macrogol	5.29	0.67	0.65	1.90	0.93	0.66	4.32	0.75	1.03	1.33	1.66	1.26	2.01	3.44	2.82
Megestrol															
Mycophenolic ac.	0.05		0.01	0.06			0.01		4.97		0.01		0.01		
Paracetamol	980	119	79.8	200	78.5	112	283	30.4	29.9	53.5	171	54.8	154	38.0	137
Prednisone	0.07	0.19	0.20	0.11	0.08	0.14	0.09	0.10	0.11	0.08	0.04	0.17	0.05	0.08	0.14
Pregabalin	1.19	1.89	3.10	0.34	1.29	1.27	2.60	2.02	1.46	1.91	0.44	0.41	0.45	0.39	0.67
Quetiapine															
Rosuvastatin			0.01		0.03						0.01	0.01	0.01		
Sulfamethoxazole	0.05														
Tiotropium						0.07						0.01			
Trazodone															
Valsartan		0.01	0.32	0.01		0.06		0.01					0.01		4.25
Vildagliptin	0.24	0.74	0.88	0.28	0.50	1.06	0.55	1.23	0.48	0.17	0.38	0.64	0.08	0.48	0.53

Table S5. Concentration (µg/L) of pharmaceuticals in FR 2. Shaded areas indicate values below MDL.

	FR 2											
	03/07/2017		04/07/2017			05/07/2017			06/07/2017		07/07/2017	
**Compounds**	**9h**	**15h**	**9h**	**13h**	**17h**	**9h**	**13h**	**15h**	**9h**	**15h**	**9h**	**15h**
Dichlorobenzyl alcohol	0.10	0.09	0.07	0.07				0.07			0.07	
Amoxicillin								0.39				

Amylmetacresol						0.29						
Acetylsalicylic acid	3.64	3.18	4.51	4.57	4.51	3.07	3.15	3.43	1.91	2.30	1.72	2.43
Atorvastatin			0.01	0.01	0.01	0.01	0.01			0.01	0.01	
Bicalutamide												
Budesonide												
Caffeine	10.3	7.37	4.49	3.95	4.98	12.0	10.8	8.14	7.35	4.39	6.29	4.02
Carbamazepine	1.96	1.78	0.61	0.44	0.93	2.90	2.76	2.06	1.92	0.92	1.18	0.48
Chlormethiazole												
Chlorpheniramine												
Clarithromycin												
Cloperastine												
Cyclophosphamide	0.01	0.01	0.03	0.02	0.02	0.03	0.04	0.03	0.02	0.03	0.03	0.03
Dextromethorphan												
Diclofenac	0.02	0.02	0.04	0.01	0.02	0.06	0.19	0.04	0.04	0.02	0.03	0.01
Donepezil						0.01	0.01					
Dutasteride												
Escitalopram									0.01			
Estrone	0.05	0.06	0.12	0.07	0.06	0.04	0.21	0.21	0.24	0.12	0.15	0.13
Ezetimibe												
Fluticasone												
Furosemide	0.04	0.02	0.03	0.01	0.01	0.02	0.02	0.03	0.02	0.02	0.02	0.02
Gabapentin												
Ibuprofen	0.35		0.13									
Ifosfamide	1.95	3.45	1.04	1.66	2.85	6.07	4.82	2.49	2.25	2.92	3.55	2.11
L-Ascorbic Acid	2.06	2.99	0.99	1.44	1.55	2.74	2.74	2.18	2.76	1.26	3.69	1.82
Levetiracetam	3.76	3.23	1.20	0.99	1.99	3.49	3.98	2.45	2.84	1.34	1.27	0.48
Levofloxacin												
Lidocaine												
Macrogol	5.48	5.98	0.45	0.33	0.59	1.97	1.54	1.12	1.96	0.52	0.52	0.23
Megestrol												
Mycophenolic ac.												
Paracetamol	21.6	16.4	6.14	4.04	4.28	5.67	6.54	5.74	6.80	9.43	3.57	9.72
Prednisone			0.03									
Pregabalin												
Quetiapine												
Rosuvastatin												
Sulfamethoxazole												
Tiotropium												
Trazodone												
Valsartan	0.28	0.26	0.36	0.16	0.18	0.12	0.21		0.59	0.44	0.21	0.17
Vildagliptin												

Table S6. Concentration (µg/L) of pharmaceuticals in PT 1. Shaded areas indicate values below MDL.

	PT 1							
	27/06/2017		28/06/2017		29/06/2017		30/06/2017	
**Compounds**	**9h**	**16h**	**9h**	**16h**	**9h**	**16h**	**9h**	**16h**
Dichlorobenzyl alcohol			0.17	0.10	0.32	0.11	0.56	0.09
Amoxicillin								
Amylmetacresol	14.4	18.1	2.16		11.23	1.62	3.58	
Acetylsalicylic acid	0.51	2.32	0.58	1.12	2.91	1.09	0.92	0.51
Atorvastatin								
Bicalutamide								
Budesonide					0.07			
Caffeine	2.32	3.25	3.67	10.1	2.51	13.3	44.5	1.08
Carbamazepine	0.11	0.08	1.58	0.02	0.04	0.03	0.06	0.07
Chlormethiazole								
Chlorpheniramine								
Clarithromycin								
Cloperastine								
Cyclophosphamide		0.04	0.02	0.01		0.01	0.01	0.01
Dextromethorphan								
Diclofenac	0.04	0.12		0.07	4.07	0.11		0.05
Donepezil	0.04	0.02	0.22		0.02		0.05	0.04
Dutasteride								
Escitalopram	0.09		0.04	0.06	0.06	0.04	0.04	0.06
Estrone								
Ezetimibe								
Fluticasone								
Furosemide	2.39	2.15	0.03	0.05	3.04	0.67	0.03	1.20
Gabapentin	0.13		0.26	0.08	0.41	0.26	0.03	0.12
Ibuprofen	1.52	2.85	0.44			0.41	0.15	4.57
Ifosfamide	38.2	40.8	16.2	3.61	7.45	7.00	16.3	3.10
L-Ascorbic Acid	9.56	2.52	4.74	2.62	2.92	2.48	11.8	2.66
Levetiracetam	326	22.1	16.9	10.9	2.79	11.3	2257	
Levofloxacin								
Lidocaine		0.14						
Macrogol	1.71	4.13	1.58	2.15	1.38	1.08	0.51	
Megestrol							0.01	
Mycophenolic ac.				0.01				
Paracetamol	3.12	2.05	174	21.8	4.55	8.95	0.91	0.42
Prednisone	0.04		0.09	0.10	0.09			0.05
Pregabalin	0.21	2.41			0.72		1.47	
Quetiapine	0.05	0.01	0.08		0.55	0.02	1.10	0.04

Rosuvastatin								
Sulfamethoxazole								
Tiotropium								
Trazodone	6.03	1.33	2.51	0.10	0.60	0.53	13.0	0.21
Valsartan								
Vildagliptin	0.04	0.01	0.01	0.06		0.27	0.02	0.15

Table S7. Concentration (µg/L) of pharmaceuticals in PT 2.

	PT 2							
	27/06/2017		28/06/2017		29/06/2017		30/06/2017	
**Compounds**	**9h**	**16h**	**9h**	**16h**	**9h**	**16h**	**9h**	**16h**
Dichlorobenzyl alcohol			1.18	0.12	0.10	0.12	0.08	0.08
Amoxicillin								
Amylmetacresol		4.96	1.01	1.53	0.36	1.38		2.03
Acetylsalicylic acid		1.52	0.79	9.22	2.71	0.87	1.72	1.10
Atorvastatin				0.01				
Bicalutamide								
Budesonide								
Caffeine	0.33	1.84	5.69	0.32	0.77	37.07	1.60	16.7
Carbamazepine	0.01	0.08	0.02	0.01	0.10	0.04	0.01	0.02
Chlormethiazole								
Chlorpheniramine								
Clarithromycin								
Cloperastine								
Cyclophosphamide	0.01	0.01						0.01
Dextromethorphan								
Diclofenac		0.41	0.01		0.02	0.02		0.07
Donepezil		0.18	0.04		0.01	0.05		
Dutasteride								
Escitalopram								
Estrone								
Ezetimibe								
Fluticasone								
Furosemide	0.01	2.63	0.03	18.6	0.70	1.15	0.04	1.88
Gabapentin	0.01	0.14	0.35	0.60	1.37	0.36	0.06	9.74
Ibuprofen		0.56	0.18	0.80	0.13		0.21	1.11
Ifosfamide	1.26	9.19	1.29	5.62	1.55	4.51	3.43	12.2
L-Ascorbic Acid	1.10	15.3	8.37	8.46	3.11	3.87	8.24	8.28
Levetiracetam		1.80	7.94	21.0	309	9.76	4.66	27.4
Levofloxacin								
Lidocaine								

Macrogol	0.79	0.12	0.17	0.21	2.68	1.46	0.40	0.92
Megestrol								
Mycophenolic ac.		0.01	0.01	0.01	0.01	0.01	0.01	0.02
Paracetamol	0.16	0.35	1.58	17.6	2.41	78.7	1.34	107
Prednisone								
Pregabalin								0.64
Quetiapine			0.04	0.03	0.04	0.03		
Rosuvastatin		0.27	0.42	0.19	0.36	0.66	0.03	0.70
Sulfamethoxazole		0.06		0.07	0.05	0.40	0.02	1.00
Tiotropium								
Trazodone		0.01	0.04		0.04	0.01		
Valsartan		0.07		0.04	0.03	0.04	0.01	0.21
Vildagliptin			0.02	0.18	0.03		0.07	0.35
